# Metastasis of the Mucionous adenocarcinoma of breast to the mandibular gingiva: Rare case report

**DOI:** 10.1097/MD.0000000000030732

**Published:** 2022-09-23

**Authors:** Ivana Mijatov, Aleksandra Fejsa Levakov, Aleksandar Spasić, Jelena Nikolić, Saša Mijatov

**Affiliations:** a Faculty of Medicine, University of Novi Sad; Clinic for Maxillofacial Surgery, University Clinical Center of Vojvodina Novi Sad, Novi Sad, Serbia; b Faculty of Medicine, University of Novi Sad; Pathology and Histology Centre, University Clinical Center of Vojvodina Novi Sad, Novi Sad, Serbia; c Faculty of Medicine, University of Novi Sad; Radiology Centre, University Clinical Center of Vojvodina Novi Sad, Novi Sad, Serbia; d Faculty of Medicine, University of Novi Sad; Clinic for Plastic and Reconstructive Surgery, University Clinical Center of Vojvodina Novi Sad, Novi Sad, Serbia; e University Clinical Center of Vojvodina Novi Sad, Novi Sad, Serbia.

**Keywords:** breast cancer, gingiva, mandible, metastasis, mucionous adenocarcinoma

## Abstract

Mucinous adenocarcinoma (MAC) is a rare type of cancer in which more than 50% of the tumor is composed of extracellular mucin and malignant epithelial cells. MACs account for only 1.8% of all breast cancer cases. Most breast cancers present as localized diseases and are well-differentiated. Breast MAC has a better 5- and 10-year survival rate than ductal and lobular carcinomas. Distant metastasis in breast MAC is rare, especially in the oral cavity. Only 1% of all oral malignancies present with metastases to the oral cavity. The bony structures are more involved than the soft tissues. Involvement of the oral soft tissue is rare, accounting for less than 0.1% of oral metastases.

**Case description:**

This report describes a rare case of mucinous breast adenocarcinoma with metastasis to the mandibular molar region. Diagnosis was established based on anamnesis, clinical presentation, tumor biopsy, computed tomography, mammography, and core biopsy of the breast tumor. The patient was sent to the oncology committee for breast disease where chemotherapy was indicated.

**Discussion:**

The clinical presentation of oral metastasis is not pathognomonic, and pyogenic granuloma, periodontal abscesses, sarcomas, and squamous carcinoma must be considered in the differential diagnosis. This is a rare case of oral metastasis of breast MAC, which was indicated for detection of the primary tumor.

## 1. Introduction

Tumor metastasis occurs when tumor cells detach from the primary tumor and migrate through the blood vessels, lymph vessels, or serosal surfaces. In the oral region, most metastases involve spreading of the tumor to the jawbones, especially to the mandible as the predominant site, with a rare invasion of soft tissue, predominantly the gingiva and tongue.^[1]^ Of all malignancies in the oral region, only 1% represent distant metastasis of the primary tumor localized elsewhere in the body, including the lungs and prostate, in the male population and breasts in the female population.^[2]^

Mucinous adenocarcinoma (MAC) is an uncommon type of cancer in which >50% of the tumor comprises extracellular mucin and malignant epithelial cells.^[3]^ Mucin is a high molecular weight glycoprotein consisting of oligosaccharides attached to proteins. MACs are extremely rare, accounting for approximately 1% of all cancer diagnoses.^[4]^ MAC commonly develops in the colorectal sites, breasts, and lungs.

Only 1.8% of all breast cancers are MACs.^[5]^ Most breast cancers present as localized diseases and are well-differentiated. According to the latest WHO Health Organization classification of breast tumors, mucinous carcinoma is a special type of breast cancer. Mucinous carcinomas are divided into 2 subtypes based on their cellularity.

- The pure type mucinous carcinoma.
- The mixed type mucinous carcinoma.

The pure form consists exclusively of tumor tissue, with extracellular mucin production in over 90% of tumors, whereas the mixed form contains an infiltrating ductal epithelial component without mucin.^[6]^ Mucinous breast cancer has a favorable prognosis with a low recurrence rate and incidence of lymph node metastasis. The final diagnosis and classification of mucinous breast carcinomas are mainly based on histopathological examination.

Breast MAC has a better 5- and 10-year survival rate than ductal and lobular carcinomas. Distant metastasis in breast MAC is rare, particularly in the oral cavity.^[7]^

Only 1% of all oral malignancies present with metastasis to the oral cavity. The bony structures are more involved than the soft tissues. Oral soft tissue involvement is rare, accounting for less than 0.1% of oral metastases.^[2]^ The lower jaw was more affected than the upper jaw was. The site of metastasis is usually the molar region of the mandible and the ramus. The reason for this is the hematopoietic bone marrow with good blood flow and branching of blood vessels, which makes this localization vulnerable to the accumulation of cancer cells. Metastasis can be clinically noted before tooth extraction, indicating the reason for tooth removal, or it can occur as vegetation after tooth extraction. In post-extraction detected metastasis, the symptoms can be mistaken for common conditions such as toothache, osteomyelitis, temporomandibular joint disorders, neuralgia, pyogenic granuloma, epulis etc. "Numb chin syndrome" caused by infiltration of an inferior alveolar branch of the mandibular nerve can be detected and presented with paresthesia in the area of innervation.^[1,2]^

The clinical presentation of metastatic tumors in the oral cavity can vary from a wide variety of clinical symptoms including pain, swelling, irritation, halitosis, tooth mobility, trismus, and numbness.

This study aimed to present a rare case of mucinous breast adenocarcinoma that metastasized to the mandibular molar region after tooth extraction. Informed consent was obtained from the patient for publication of this case report details.

## 2. Case report

**Figure 1.**
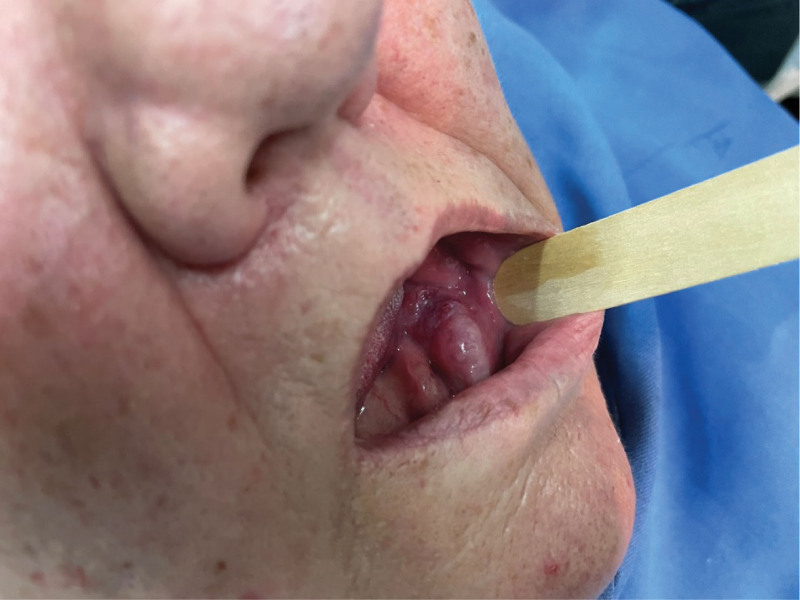
Intraoral clinical findings.

**Figure 2.**
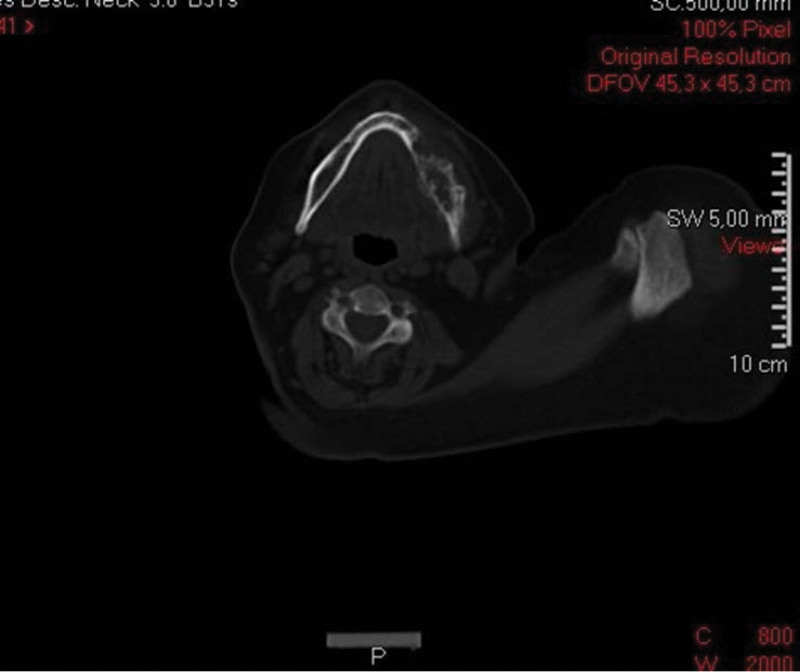
CT scan of the lower jaw. CT = computed tomography.

**Figure 3.**
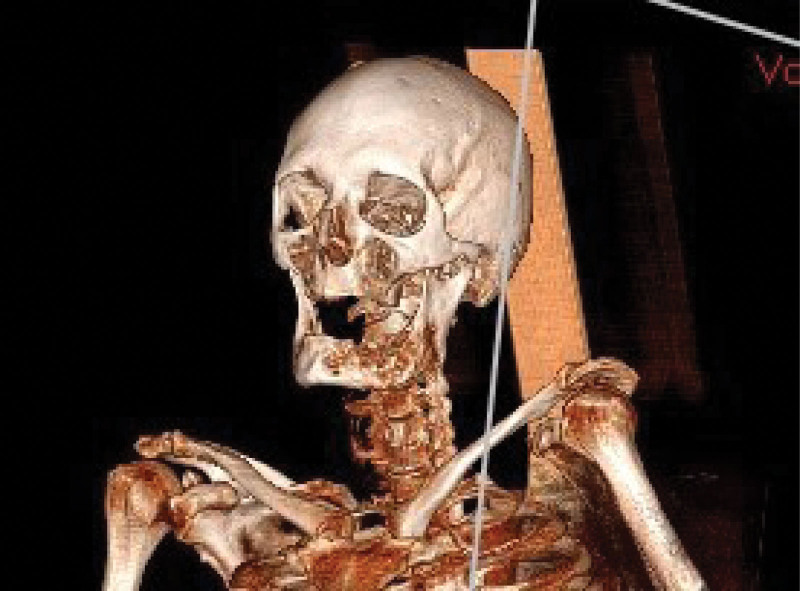
3D CT scan of jaw. CT = computed tomography.

**Figure 4.**
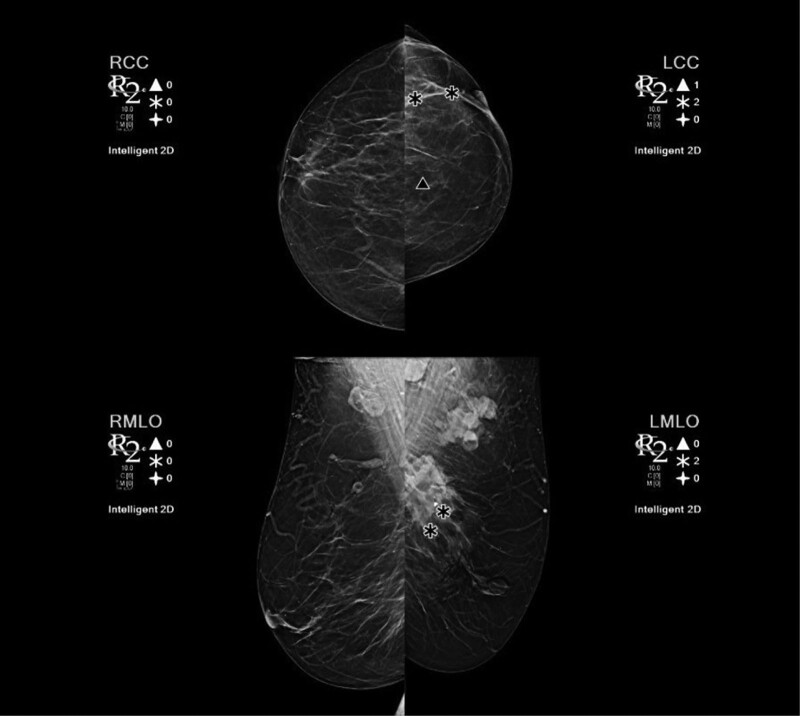
Mammography.

**Figure 5.**
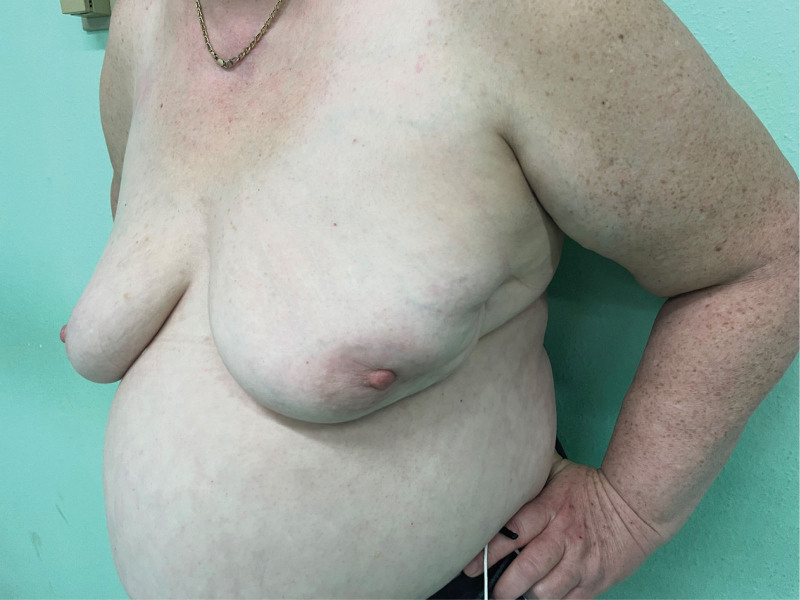
Clinical findings on the breast.

**Figure 6.**
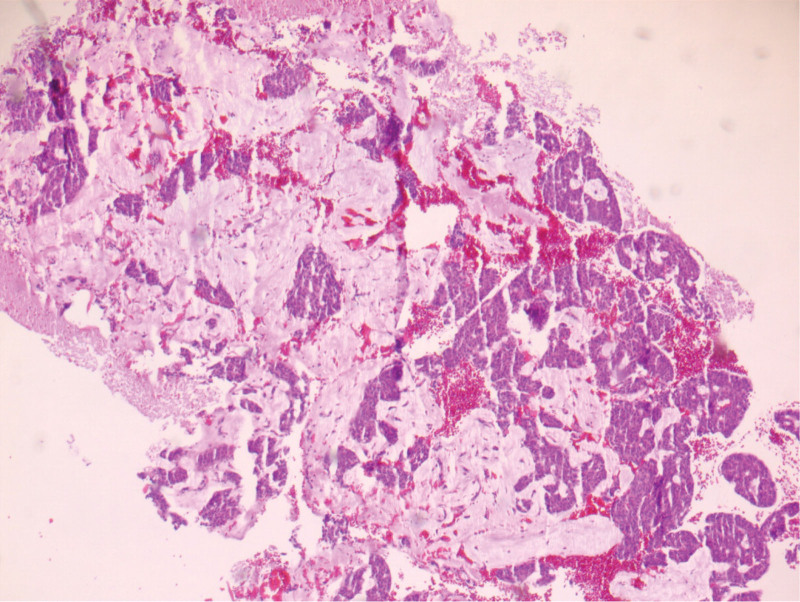
HE tumor of gingivae 5×.

**Figure 7.**
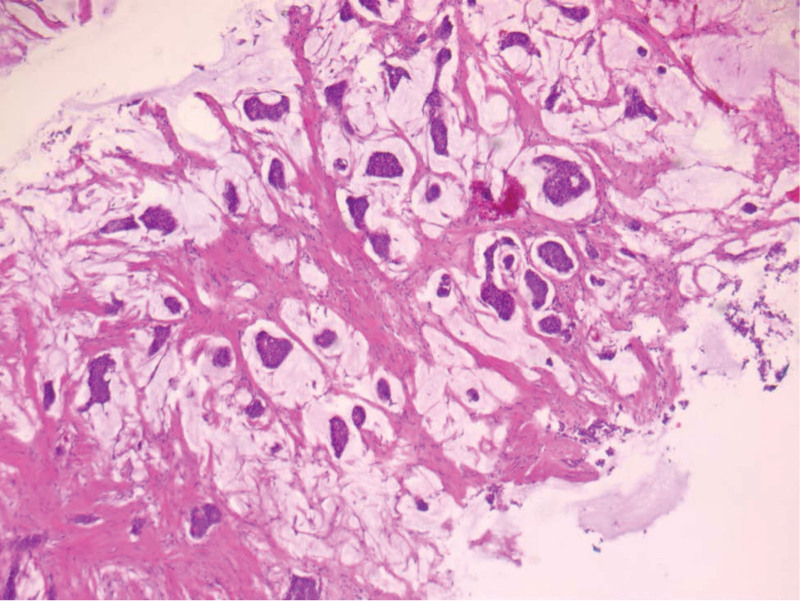
Breast tumor HE 5×.

A 58-years-old female was referred to the Clinic for Maxillofacial Surgery, University Clinical Center of Vojvodina in Novi Sad, complaining of a nonhealing post-extraction wound in the gingiva of the mandible on the left side. Three months after tooth 36 was extracted, the wound did not heal. Swelling and vegetation of the gingiva were observed in the mandibular region on the left side after the intraoral examination, and the OPT determined that the bone was destroyed (Fig. 1). The patient did not report any other complaints or illnesses. Differential diagnosis included pyogenic granuloma, squamous cell carcinoma, or some type of mesenchymal tumor of the jaw, chondrosarcoma, or osteosarcoma. An incisional biopsy of the gingival tumor was performed and the patient underwent computed tomography of the head, neck, and chest as part of the oral carcinoma TNM staging protocol (Figs. 2 and 3). Based on the computed tomography examination, a radiologist described an expansive-infiltrating tumor in the area of the mandible body with infiltration into the surrounding tissue. The size of the tumor was 60 × 30 × 40 mm. The bone was destroyed with a “sunburst” periosteal reaction and the borders were independent compared to the surrounding soft tissue. The tumor infiltrated the mylohyoid muscle, pterygoid muscles, and the anterior belly of the digastric muscle. In addition, the radiologist described expansive soft tissue changes in the left breast with dimensions of 60 × 40 × 35 mm and infiltrating lymph nodes in the area of the left axilla. Mammography, and core biopsy of the breast tumor was done (Figs. 4–6). Osteoblastic changes were also observed in the 3^rd^ thoracic spinal vertebra. The pathologist described metastatic MAC based on the tissue obtained from the gingival biopsy. Microscopic examination of the specimens revealed nests of cells floating in lakes of mucin partitioned by delicate fibrous septa containing capillary blood vessels. The cell clusters varied in shape and size with occasional tubular arrangements. In order to find the primary tumor, mammography and core biopsy of the breast tumor were done and, after pathophysiological analysis and immunohistochemistry, it was found that the primary tumor was a breast tumor and that the gingival tumor was a metastatic tumor from the breast MAC. The tumor was estrogen-(90% of cells, Allred score 8) and progesterone receptor-positive (40% of cells, Allred score 6), and the Ki67 proliferative index was 30% (30% of the nuclei of the tumor cells had a positive reaction). HER2 receptors were 1+, CK7+, CK20+, mammaglobin −, and GATA-3 + (Figs. 6 and 7). The patient was sent to the Oncology Committee for Breast Diseases, where chemotherapy was indicated with 3 cycles per anthracycline (AC) protocol, after which the effects of therapy and the inclusion of irradiation therapy for mandible metastasis were evaluated.

## 3. Discussion

Metastasis is a secondary tumor implant distant from the primary tumor site. Metastatic lesions of the oral cavity are rare and challenging for clinicians in the diagnostic sense. These lesions are not pathognomonic and can be confused with tumors of different oral locations. The kidneys, liver, gastrointestinal tract, and lungs are the most common primary sites of oral metastasis. The mandible is more often affected, with 69% to 82% of all jaw metastases occurring in the mandible.^[1]^ Metastasis in the oral region is often the first symptom of malignancy, and may indicate the discovery of a primary tumor.

Several theories explain site-specific tumor metastasis (Paget 1889 “seed and soil” theory, Zetter 1990 “mechanical theory”).^[8]^ Metastasis can be explained by a combination of 2 theories: tumor cells flow through the lymphovascular system, implant into new tissue, and grow in it, resulting in distant metastasis. Site–specific metastasis is caused by growth factors released from different tissues. The valveless vertebral venous plexus and Batson veins bypass filtration by the lungs and represent pathways for metastasis from the gastrointestinal, genitourinary, and respiratory systems to the maxillofacial region. Jawbones are mostly affected in the oral region, particularly the mandible, twice as often as the maxilla (2:1), whereas the gingiva is rarely affected.^[1,2]^ Some authors have emphasized that the duration of gingival inflammation plays an important role in the development of metastasis in this region. In our case, metastasis occurred in the region of the body of the mandible and the region of the gingiva 3 months after tooth extraction. In this case, the tooth extraction promoted metastasis.

Breast cancer specifically MAC rarely metastasizes to the oral region.

Mucinous breast adenocarcinoma is a rare malignant breast tumor that is more common in perimenopausal and postmenopausal women.^[9]^ Breast MAC has a better prognosis than other malignant breast tumors (ductal or lobular carcinoma). Metastatic diseases of the breast MAC are rare. The largest case series reported rates between 12% and 14% for nodal metastasis and only 2% for distant metastasis at the time of surgery.^[5,10]^

Breast MAC treatment includes surgical treatment supported by adjuvant chemotherapy, radiotherapy, and additional adjuvant endocrine therapy for hormone receptor-positive tumors.^[11]^ Advanced stages of pure breast MAC are very rare, and mixed types of breast MAC are more invasive and often associated with local metastasis to axillary lymph nodes.^[11,12]^ Our case represents a rare case of advanced pure breast MAC with distant metastasis to vertebral and mandibular bodies. Patient non-cooperation and disease denial were the main reasons for such a condition, considering that breast screening is an easily accessible and simple screening method.

To the best of our knowledge, this is the first reported case of oral metastasis of a mucinous breast adenocarcinoma. Involvement of the oral tissue in ductal and lobular breast carcinoma has been described, but oral metastasis of MAC is rare.

Patients with oral metastases often show metastasis to other sites. Our patient had bone metastasis in the thoracic spine.

Patients with oral metastases are usually previously treated because of their primary tumors. In our case, the patient did not receive any treatment and the primary tumor was discovered during the diagnosis of an oral lesion. The reason for this is paramedical: the patient sensed the existence of the breast tumor but denied to the best of our knowledge, this is the first reported case of oral metastasis of mucinous breast adenocarcinoma. Involvement of the oral tissue in ductal and lobular breast carcinoma has been described, but oral metastasis of MAC is rare.

Patients with oral metastasis often show metastasis it out of fear.

The prognosis of breast MAC is better than that of other breast carcinomas.^[13,14]^ In this case, the advanced disease could not be treated surgically but only by radiotherapy and chemotherapy. The disease stage affects disease prognosis. The prognosis of patients with oral metastatic lesions is poor and the expected median survival is several months.^[15]^ The treatment of advanced disease is mainly palliative.

## 4. Conclusion

Oral metastasis of breast tumors is rare, particularly in MACs. The clinical presentation of oral metastasis is not pathognomonic, and pyogenic granuloma, periodontal abscesses, sarcomas, and squamous carcinoma must be considered in the differential diagnosis. Anamnesis and good clinical examination are important for proper diagnosis. Despite the advances in breast cancer screening, sporadic cases of advanced breast cancer continue to occur. This is a rare case of oral metastasis of breast MAC, as indicated by detection of the primary tumor. When treating such patients, a multidisciplinary approach, careful and precise examination, and high degree of clinical suspicion are required.

## Author contributions

**Conceptualization:** Ivana Mijatov, Jelena Nikolić.

**Investigation:** Aleksandra Fejsa Levakov, Aleksandar Spasić.

**Resources:** Ivana Mijatov, Aleksandra Fejsa Levakov, Aleksandar Spasić.

**Software:** Aleksandar Spasić, Saša Mijatov.

**Validation:** Ivana Mijatov.

**Visualization:** Saša Mijatov.

**Writing – original draft:** Ivana Mijatov.

**Writing – review & editing:** Ivana Mijatov.
